# Upregulation of IFNɣ-mediated chemokines dominate the immune transcriptome of muscle-invasive urothelial carcinoma

**DOI:** 10.1038/s41598-021-04678-7

**Published:** 2022-01-13

**Authors:** Ekaterina Olkhov-Mitsel, Anjelica Hodgson, Stan K. Liu, Danny Vesprini, Jane Bayani, John M. S. Bartlett, Bin Xu, Michelle R. Downes

**Affiliations:** 1 Division of Anatomic Pathology, Laboratory Medicine and Molecular Diagnostics, Sunnybrook Health Sciences Centre, 2075 Bayview Avenue, Toronto, ON M4N 3M5 Canada; 2 Department of Pathology, Laboratory Medicine Program, University Health Network - Toronto General Hospital, Toronto, ON Canada; 3 Laboratory Medicine and Pathobiology, University of Toronto, Toronto, ON Canada; 4 Radiation Oncology, Sunnybrook Health Sciences Centre, Toronto, ON Canada; 5 Diagnostic Development, Ontario Institute for Cancer Research, Toronto, ON Canada; 6 Department of Pathology, Memorial Sloan Kettering Cancer Center, New York City, NY USA

**Keywords:** Tumour immunology, Urological cancer, Translational research

## Abstract

Tumor inflammation is prognostically significant in high-grade muscle-invasive bladder cancer (MIBC). However, the underlying mechanisms remain elusive. To identify inflammation-associated immune gene expression patterns, we performed transcriptomic profiling of 40 MIBC archival tumors using the NanoString nCounter Human v.1.1 PanCancer Panel. Findings were validated using the TCGA MIBC dataset. Unsupervised and supervised clustering identified a distinctive immune-related gene expression profile for inflammation, characterized by significant upregulation of 149 genes, particularly chemokines, a subset of which also had potential prognostic utility. Some of the most enriched biological processes were lymphocyte activation and proliferation, leukocyte adhesion and migration, antigen processing and presentation and cellular response to IFN-γ. Upregulation of numerous IFN-γ-inducible chemokines, class II MHC molecules and immune checkpoint genes was detected as part of the complex immune response to MIBC. Further, B-cell markers linked to tertiary lymphoid structures were upregulated, which in turn is predictive of tumor response to immunotherapy and favorable outcome. Our findings of both an overall activated immune profıle and immunosuppressive microenvironment provide novel insights into the complex immune milieu of MIBC with inflammation and supports its clinical significance for predicting prognosis and immunotherapeutic responsiveness, which warrants further investigation. This may open novel opportunities to identify mechanisms for developing new immunotherapeutic strategies.

## Introduction

Bladder cancer is the ninth most prevalent malignancy with substantial mortality rates and recurrence risk^1^. Approximately 25% of patients are diagnosed with muscle-invasive disease (MIBC) with an associated 5-year survival of 36–48%^2^. Urothelial carcinoma (UC), the most frequent histologic type of bladder cancer, is known to be immunogenic and responsive to immunotherapy including intravesical Bacillus-Calmette Guerin and immune checkpoint inhibitors (ICIs)^3^. Although ICIs are very effective in a small proportion of UCs, many patients either do not respond or do not have a durable response highlighting the need to better understand predictors of response and disease biology^4^.

We and others have previously shown that the presence of inflammation, observed in biopsies and excision specimens, is associated with malignant progression, efficacy of anti-cancer therapies, and patient outcome^5–10^. However, the underlying signaling pathways and immunobiological mechanisms for this association and their implications are not well understood.

Emerging data suggests that enrichment in tumor-infiltrating CD8 + T cells and expression of immune genes such as *IFN-γ, CXCL9*, and *CXCL10* is an indicator for immunotherapy response^11–13^. Further, low Neutrophil-to-lymphocyte ratio, macrophage infiltration and human leukocyte antigen (HLA) class I and II expression has been shown to predict tumor response to ICIs^14–16^. However, a cohesive picture of the immune response to MIBC and the underlying molecular pathways are still lacking. Future progress in MIBC therapeutic approaches will require a strong understanding of the immunology of this disease. Therefore, research into the immune landscape of MIBC may lead to the development of more precise prognostication, patient stratification and personalized treatment decisions. In this regard, transcriptomic data is increasingly being used to determine the tumor immune microenvironment and these methods have been shown to be very efficient and reliable^17^. Targeted gene expression panels, such as NanoString panels, have the potential to assess the tumor microenvironment and immune milieu of MIBCs comprehensively and ascertain the inflammatory status of a tumor by quantifying chemokines, cytokines, cell surface proteins and transcription factors along with immune checkpoints.

In the current study, we investigated gene expression profiles of immune-related genes (IRGs) as detected by NanoString technology to generate knowledge about the tumor immune milieu in highly inflamed versus low-inflamed MIBCs and better understand the potential immunobiological mechanisms for the association of inflammation with clinical outcome.

## Results

### Sunnybrook high-grade urothelial carcinoma cohort

**Figure 1:**
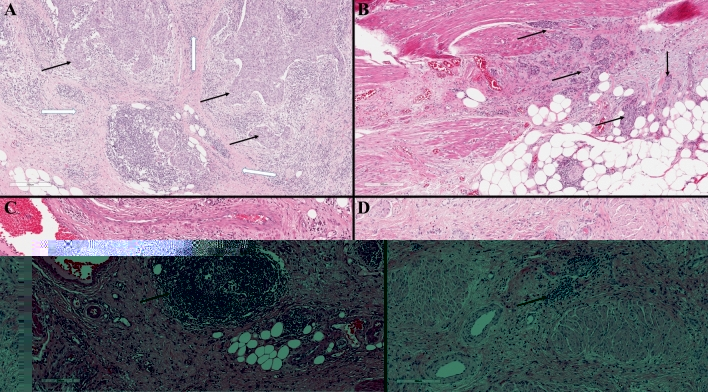
Haemotoxylin and eosin images showing spectrum of inflammation in high grade muscle invasive urothelial carcinoma. (**A**) Low power image showing carcinoma-stroma interface with high invasive front inflammation (black arrows indicate invading carcinoma, white arrows the stroma), (**B**) low power image with minimal invasive front inflammation (black arrows highlight areas of carcinoma), (**C**) well defined lymphoid structure with germinal centre in keeping with a tertiary lymphoid structure (indicated by black arrow), (**D**) loose collection of lymphocytes comprising a lymphoid aggregate (indicated by black arrow).

The clinicopathologic characteristics of the MIBC cohort are listed in Table 1. Patients with high inflammation intra-tumorally also had high inflammation at the invasive front. There was a significant association of tertiary lymphoid structures (TLS) with high inflammation (19/20) and only one case of low inflammation containing TLS (*P* < 0.0001, Fig. 1). Four low inflammation tumors had small lymphoid aggregates lacking defined germinal centers. These were remote from the tumor edge in the perivesical adipose tissue.

**Table 1:** Clinicopathologic characteristics of the Sunnybrook NanoString cohort.

	Total (N = 40)	Low Inflammation (N = 20)	High Inflammation (N = 20)	*P* Value
	N (%)	N (%)	N (%)	
**Age (years)**				0.205
Mean (Range)	69.8 (33–88)	72.1 (50–88)	67.6 (33–84)	
**Sex**				1.000
Male	28 (70)	14 (70)	14 (70)	
Female	12 (30)	6 (30)	6 (30)	
**CIS**				0.525
Present	18 (45)	8 (40)	10 (50)	
Absent	22 (55)	12 (60)	10 (50)	
**pT Category**				0.065
pT2	1 (2.5)	0	1 (5)	
pT3	29 (72.5)	12 (60)	17 (85)	
pT4	10 (25)	8 (40)	2 (10)	
**Margins**				0.018
Negative	27 (67.5)	10 (50)	17 (85)	
Positive	13 (32.5)	10 (50)	3 (15)	
**Lymphovascular invasion**		0.013		
Absent	11 (27.5)	2 (10)	9 (45)	
Present	29 (72.5)	18 (90)	11 (55)	
**Lymph Nodes**				0.414
Negative	25 (62.5)	11 (50.0)	14 (70)	
Positive	12 (30.0)	9 (40.9)	5 (25)	
N/A	3 (7.5)	2 (9.1)	1 (5)	
**Event-free survival**				0.003
Relapse-free	10 (25.0)	1 (5)	9 (45)	
Relapse and/or death	30 (75.0)	19 (95)	11 (55)	
**Follow-up time (months)**				
Mean (Range)	25.1 (0–206)	9.5 (0–43)	40.6 (1–206)	0.023
**Tertiary lymphoid structures**				
Present	20 (50)	1 (5)	19 (95)	< 0.001
Absent	20 (50)	19 (95)	1 (5)	

*CIS* Carcinoma In Situ, *pT* Pathologic T stage, *N/A* not available.

### Global immune gene expression patterns of high-grade urothelial carcinomas

To characterize inflammation-associated immune gene expression patterns in high versus low inflammation MIBC, NanoString profiling coupled to the nCounter human PanCancer immune panel was utilized to interrogate the mRNA expression profiles. In total, expression of 730 mRNAs from 24 different immune cell types related to both innate and adaptive immune responses were screened. Of these, 532 (72.9%) IRGs were expressed above background threshold levels.

**Figure 2:**
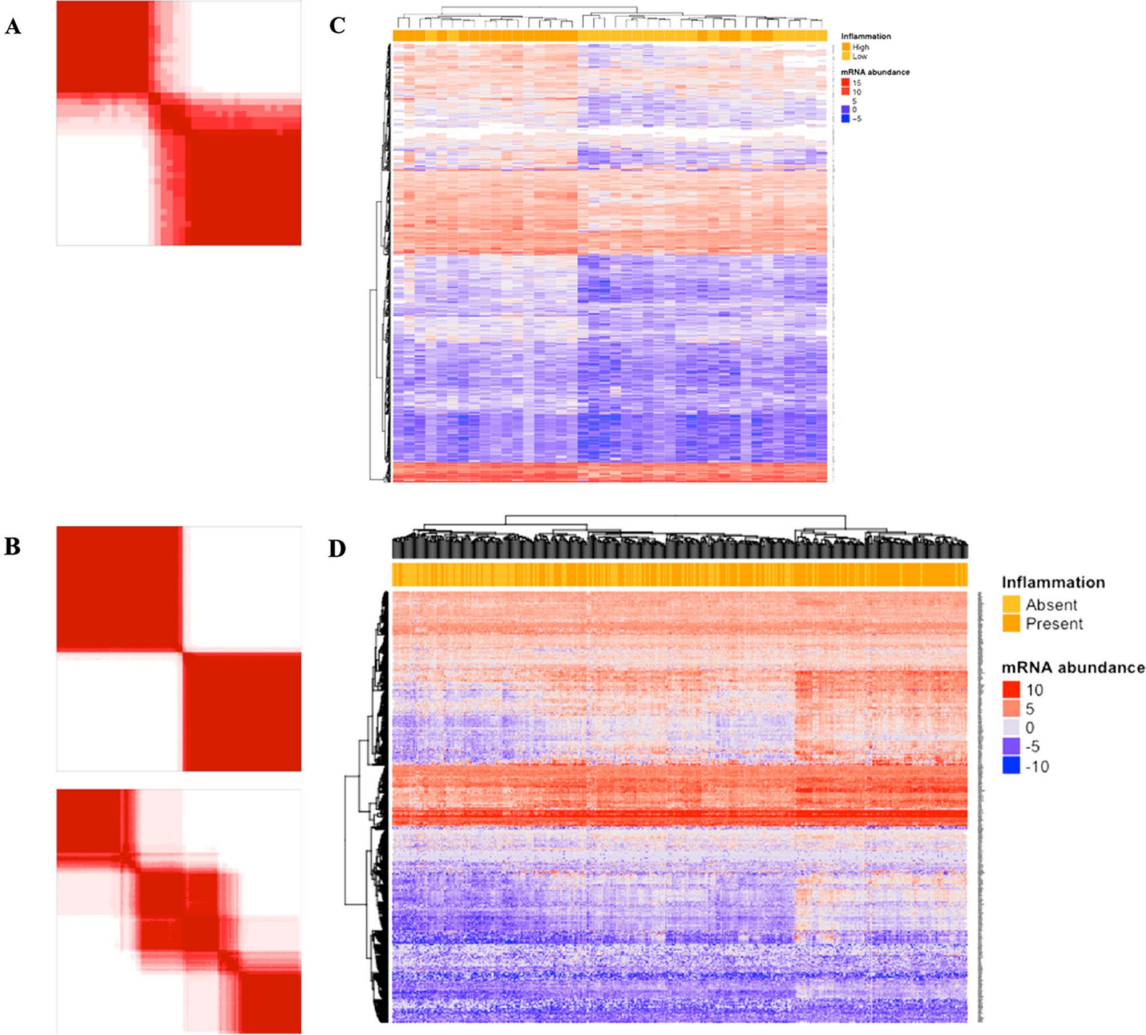
Consensus Matrix for two unsupervised clusters for (**A**) Nanostring discovery cohort and (**C**) TCGA cohort of MIBCs. Consensus matrices represented as heatmaps for the chosen optimal cluster number (k = 2) for Nanostring discovery and TCGA cohorts, respectively. Patient samples are both rows and columns, and consensus values range from 0 (never grouped together, indicated in white) to 1 (always clustered together, indicated in red). (**B**) Hierarchical clustering (Euclidean correlation) of normalized abundance levels of 730 mRNAs on the NanoString human PanCancer immune panel from Nanostring discovery cohort and (**D**) TCGA cohort of MIBCs.

Unsupervised hierarchical clustering of gene expression levels using Euclidean correlation segregated the 40 MIBCs into two clusters, representing different immune profiles (Fig. 2A–B). Further, K-Means consensus clustering analysis also yielded the same two clusters, suggesting that two robust immune expression subtypes exist in our cohort. Cluster I consisted of primarily high inflammation cases (15/17, 88.2%) while cluster II consisted primarily of cases with low inflammation (18/23, 78.3%).

### Differentially expressed immune related genes associated with inflammation

**Figure 3:**
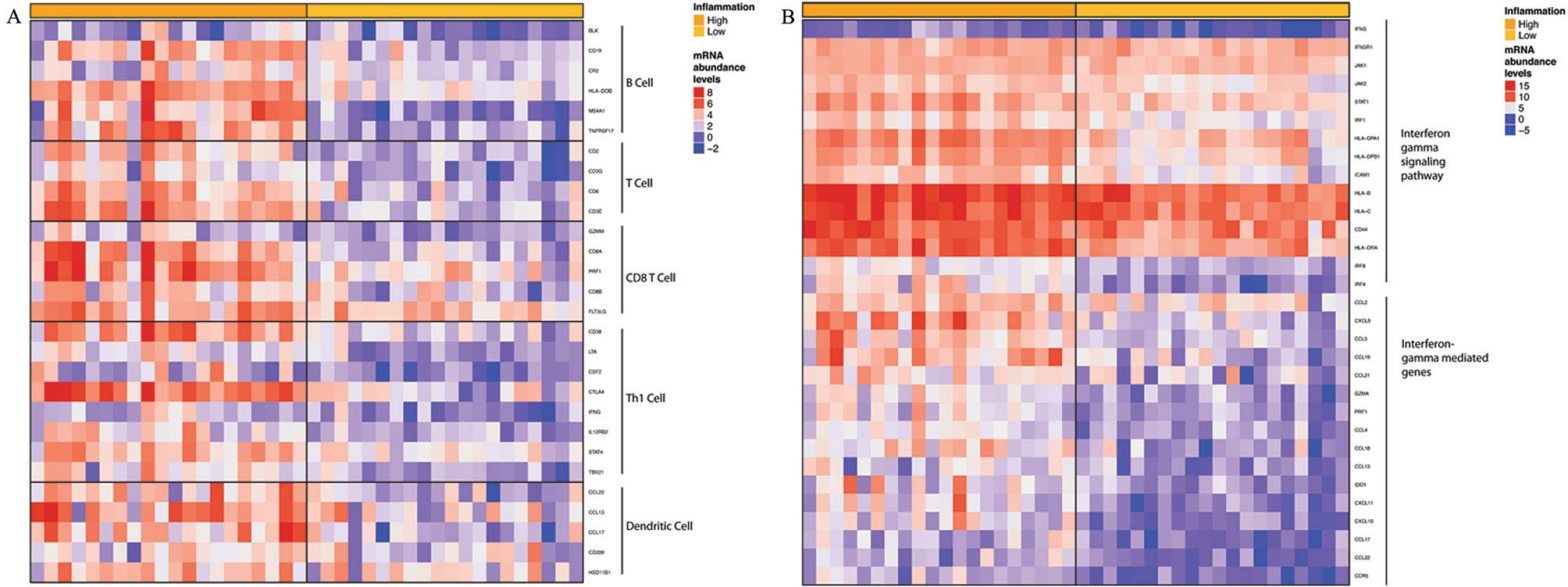
Expression pattern of representative genes in (**A**) immune cells. Top to bottom: B cell, T cell, CD8, Th1 and dendritic cell expression clusters as defined by the NanoString human PanCancer immune panel. (**B**) Unclustered expression levels of IFN-γ signaling pathway and downstream response genes among high- and low- invasive front inflammation muscle invasive urothelial carcinomas in the NanoString discovery cohort.

To investigate the inflammation-associated modulation in immune gene expression patterns between high and low inflammation, linear regression analysis with multiple-testing correction was utilized. As expected, the immune signature of high inflammation tumors was characterized by significant (FDR *P* ≤ 0.05) over twofold altered expression of 149 genes associated with key immune response pathways, 99.3% of which were upregulated. The top 25 most differentially expressed genes are listed in Table 2 and these included the TLS marker *CXCL13*, B-cell related genes (*CD19, CD79, TNFRSF17, POU2AF1* and *PIK3CD*), T-cell lineage and T-effector genes (*CD2, CD3D, CD3E, CD6, CD4, CD8, IL2RG* and *CD44*) as well as genes expressed on T-regulatory cells (*FOXP3, CTLA4 and ICOS*). The leukocyte common antigen, *CD45*, was also significantly upregulated in high inflammation tumors. Assessment of the expression levels of B-cell, T-cell, CD8 T-cell, Th1 cell and dendritic cell markers also showed upregulation of these genes in high inflammation tumors (Fig. 3A).

**Table 2:** Top 25 differentially expressed genes in high- versus low- inflammation muscle invasive urothelial carcinomas in Sunnybrook NanoString cohort and their differential expression between carcinomas with- versus without- immune infiltrates in the TCGA cohort.

mRNA	Sunnybrook NanoString Cohort		TCGA Cohort	
	Linear fold change	BH-FDR *P* Value	Linear fold change	BH-FDR *P* Value
CXCL13	16.7	< 0.001	6.8	< 0.001
CXCL10	12.6	< 0.001	5.6	< 0.001
CD79A	11.4	< 0.001	5.2	< 0.001
CCL5	11.1	< 0.001	2.9	< 0.001
CCL19	11.0	< 0.001	5.2	< 0.001
GZMK	10.8	< 0.001	3.6	< 0.001
SLAMF7	10.4	< 0.001	2.2	< 0.001
CD27	10.0	< 0.001	3.0	< 0.001
CXCL9	9.6	< 0.001	6.9	< 0.001
CHIT1	9.5	< 0.001	2.4	< 0.001
TNFRSF17	9.5	< 0.001	2.5	< 0.001
IDO1	9.3	< 0.001	5.3	< 0.001
CD79B	8.9	< 0.001	2.1	< 0.001
CD22	8.9	< 0.001	2.7	< 0.001
IRF4	8.8	< 0.001	3.9	< 0.001
POU2AF1	8.8	< 0.001	2.9	< 0.001
CXCL11	8.5	< 0.001	5.5	< 0.001
S100A8	8.4	0.027	3.3	< 0.001
ITK	7.9	< 0.001	3.3	< 0.001
CD8A	7.9	< 0.001	3.3	< 0.001
CD3D	7.7	< 0.001	3.2	< 0.001
BTLA	7.2	< 0.001	2.3	< 0.001
LTB	7.1	< 0.001	3.1	< 0.001
CCL17	7.1	< 0.001	1.5	0.004
CTLA4	6.7	< 0.001	3.9	< 0.001

BH-FDR, Benjamini–Hochberg procedure to decreases the false discovery rate; TCGA, The Cancer Genome Atlas.

Numerous inflammatory mediators including chemokines and chemokine receptors (*CCL2, CCL3, CCL5, CCL13, CCL17, CCL22, CXCR4, CXCL9, CXCL10* and *CXCL11*) were upregulated in high inflammation tumors. However, the expression of chemokine receptor *CXCR3*, expressed on tumor-infiltrating cytotoxic T-cells, was not linked to inflammation. Similarly, interferons and cytokines such as *IL1, IL4, IL6, IL10, IL12, IL13, IL18, CSF, IFNA, MIF, TNFα* and *TGFB* were not differentially expressed between high and low inflammation tumors.

Notably, genes mediated by IFN-γ, a key cytokine in the tumor microenvironment were also significantly upregulated in high inflammation (*STAT1, CCR5, CXCL9, CXCL10, CXCL11, CCL2, CCL4, CCL5, CCL13, CCL17, CCL18, CCL19, CCL21, CCL22, IDO1, PRF1, IRF4, IRF8, GZMA, HLA-DRA, HLA-DMA*, Fig. 3B). Similarly, the tumor necrosis factor receptor superfamily (*TNFRSF*) was significantly upregulated in high inflammation tumors, including *CD27, CD40, FAS, TNFRSF13C, TNFRSF17, TNFRSF18, TNFRSF1B, TNFRSF4, TNFRSF9*.

Lastly, the immune checkpoint genes (ICGs) *CTLA-4, PD-L1, PD-2, IDO1* and *LAG3* were significantly upregulated in high inflammation tumors while *PD1* expression was below threshold of detection in tumors with low inflammation.

### Pathway analysis

To determine the inflammation-associated immunobiological pathways governing the differences between low and high inflammation, g:Profiler functional enrichment analysis was utilized. At FDR corrected *P* ≤ 0.05, we found that upregulated transcripts were significantly enriched for 85 GO biological processes, 40 KEGG pathways and 21 Reactome pathways (Supplemental Fig. 1). The most enriched biological processes distinguishing high from low inflammation were related to lymphocyte activation and proliferation, leukocyte adhesion and migration, cellular response to tumor necrosis factor, antigen processing and presentation, cellular response to IFN-γ, neutrophil activation, positive regulation of ERK1 and ERK2 cascade and calcium ion transport. The top overrepresented pathways included cytokine-cytokine receptor interaction, hematopoietic cell lineage, signaling by Interleukins, Th1, Th2 and Th17 cell differentiation, chemokine signaling, neutrophil degranulation, costimulation by the CD28 family, NF-kappa B signaling pathway, Jak-STAT signaling pathway, antigen processing and presentation and T cell receptor signaling pathway (Supplemental Fig. 1).

### Prognostic Immune-related mRNAs

Cox proportional hazards model was used to assess whether DEG between tumors with high and low invasive front (FDR-corrected *P* ≤ 0.001, n = 71) are prognostically significant for EFS. To test this effect, patients were median-dichotomized to low and high expression based on the mRNA abundance levels of each DEG. The list of genes, hazard ratios, 95% confidence intervals (CI), *P* values and BH corrected p-values (adjusted for multiple testing) are listed in Table 3. We identified 13 genes differentially expressed between high and low inflammation tumors as significant potential predictors of EFS.

**Table 3:** Univariate Cox regression analysis of differentially expressed genes between with high- versus low- invasive front for predicting EFS in muscle invasive urothelial carcinomas treated by cystectomy.

Gene Symbol	HR	95% CI		BH-FDR* P* Value
TNFRSF17	4.49	1.89	10.67	0.042
CD38	3.72	1.68	8.21	0.042
CSF2RB	3.39	1.55	7.40	0.044
POU2AF1	3.32	1.50	7.32	0.044
CD79B	3.28	1.41	7.58	0.044
SLAMF7	3.16	1.43	6.97	0.044
IRF4	3.06	1.40	6.67	0.044
CXCL13	3.01	1.38	6.58	0.044
PIK3CG	3.01	1.38	6.58	0.044
CD2	2.96	1.36	6.43	0.044
IRF8	2.84	1.31	6.15	0.048
IL10RA	2.77	1.29	5.92	0.048
ITGB2	2.74	1.29	5.80	0.047

*HR* Hazard Ratio, *CI* Confidence Interval, *BH-FDR* Benjamini–Hochberg procedure to decreases the false discovery rate.

### TCGA data

To validate the gene expression signatures we identified in association with high and low inflammation, we performed an immune transcriptomic analysis of the immune gene expression profiles of 730 genes derived from the NanoString™ panel utilized for our Sunnybrook cohort. The TCGA cohort consisted of 411 chemotherapy-naïve, muscle-invasive, high-grade urothelial tumors.

Unsupervised hierarchical clustering of gene expression levels using Euclidean correlation segregated the 411 TCGA high-grade urothelial carcinomas into two large clusters, representing different immune profiles (Fig. 2C–D). Cluster I could also be subdivided into two sub-clusters (Cluster Ia and Ib). KMeans consensus clustering analysis revealed that the TCGA cohort can be more robustly segregated into two compared to three clusters (Fig. 2C). Cluster I consisted of approximately equal distribution of cases with and without inflammatory infiltrate (146/287 49% and 141/287 51%, respectively). This cluster can be further subdivided into Cluster Ia, consisting primarily of cases without inflammatory infiltrate (74/102, 73%) and Cluster Ib; 39% (72/185) without and 61% (113/185) with inflammatory infiltrate. Cluster II consisted primarily of cases with inflammatory infiltrate (106/124, 85%).

Linear regression analysis with multiple-testing correction revealed that cases with inflammatory infiltrate were characterized by significant (FDR *P* ≤ 0.05) over twofold altered expression of 221 genes associated with key immune response pathways, 98.6% of which were upregulated. Among these, 112 were also significantly (FDR *P* ≤ 0.05) over twofold altered in our NanoString Sunnybrook cohort. Additional 32 genes were significantly (FDR *P* ≤ 0.05) altered in both Sunnybrook and TCGA cohorts, but did not reach twofold change in the latter. Therefore, 96.6% (144/149) of DEGs in our NanoString Sunnybrook cohort were validated in the TCGA cohort. Comparison of the fold change and FDR P-values for the top 25 mRNAs is presented in Table 2.

Functional enrichment analysis with g:Profiler validated that immunobiological pathways governing the differences between cases with and without inflammatory infiltrate in the TCGA cohort are largely similar to the pathways that differentiate between low and high inflammation in the Sunnybrook NanoString cohort (Supplemental Fig. 1).

## Discussion

Multigene immune signatures represent a robust means of capturing the key players in MIBC tumor-associated inflammation. In the current study, we used a NanoString immune profiling panel to analyze and characterize inflammation-associated immune gene expression patterns in high inflammmation versus low-inflammation MIBCs. Unsupervised hierarchical clustering analysis identified two clusters characterized by distinctive immune profiles. We found that those of the high-inflammation cluster had a higher expression of most immune cell markers, including Th1 cells, CD8 + and B cells. Further, numerous chemokines, which play a crucial role in modulating anti-tumor immune response, were also up-regulated in high inflammation tumors. Conversely, the low inflammation cluster had a relatively lower abundance of immune cell markers. We validated our findings and obtained similar results analyzing the TCGA MIBC dataset. Analyzing DEG between low- and high- inflammation tumors in our cohort, we identified a signature of 149 inflammation-associated IRGs, a subset of which also had a good ability to predict EFS.

Upregulation of *CCL4, CCL5, CXCL9, CXCL10* and *CXCL11* in high inflammation MIBC is noteworthy, as their presence in tumors promotes T cell infiltration, a key mechanism in antitumor immunity and could explain improved patient outcome^18^. The upregulation of these chemokines is induced by IFN-γ, secreted by Th1 cells^19^. Indeed, Th1-associated markers were upregulated in high inflammation tumors. Moreover, expression of a number of cytotoxic markers induced by IFN-γ, namely *granzymes A*, *B* and *K* as well as *PRF1* was also elevated in high inflammation MIBC, consistent with the presence of activated cytotoxic T cells together with Th1 cells in the tumors of these patients^9^. Further, we detected the upregulation of numerous additional IFN-γ-inducible chemokines and class II MHC molecules associated with anti-tumor inflammation and improved patient outcome^20^. This is in agreement with previous studies that have shown that “hot” tumor microenvironment characterized by T cell infiltration and pro-inflammatory cytokine production present higher response rates to immunotherapy compared to infiltration-excluded or “cold” tumors^21^. Further, it has been reported that patients with high immune infiltration and IFN-γ–related mRNA expression signature correlated with a clinical benefit^22^.

Of note, the IFN-γ–inducible chemokines *CXCL9, CXCL10* and *CXCL11* activate the chemokine receptor *CXCR3*, a variant transcript of which has been associated with response to neoadjuvant chemotherapy^23^. It is proposed as one of the potential mechanisms underlying the increased responsiveness of the immune-infiltrated basal subtype of MIBC to neoadjuvant chemotherapy^24^. However, the expression of *CXCR3* was not linked to inflammation in our cohort, possibly because we did not analyze the expression of the individual *CXCR3* isoform subpopulations^25^. Further research into the relevance of *CXCR3* isoforms relative to the inflammatory tumor milieu for the response to chemotherapy is needed to elucidate the role of the immune system in the response to chemotherapy in MIBC.

High inflammation MIBC tumors also showed significant upregulation of the IFN-γ-inducible ICGs *IDO1* and *PD-L1* as well as the ICGs *CTLA-4* and *LAG3*. The stimulation of these ICGs likely facilitates MIBC pro-tumor immune tolerance, suggesting that immune checkpoint blockade therapies could be more suitable for MIBC patients with high- compared to low- inflammation. This is currently being investigated in a phase II clinical trial titled DUTRENEO of durvalumab and tremelimumab versus chemotherapy as a neoadjuvant approach to MIBC treatment of patients selected based on IFN-γ immune signature^26^.

In addition to IFN-γ inducible genes, our analysis identified that *CXCL13*, a B-cell chemoattractant, and *CD79A*, part of the B-cell receptor signaling complex, are among the top most differentially expressed genes between high- and low- inflammation MIBCs. Expression of both *CXCL13* and *CD79A* has been linked to TLS, which in turn is predictive of tumor response to immunotherapy and favorable outcome in many tumor types, including MIBC^27–30^. Importantly, recent data reported a correlation between *CXCL13* expression and response to ICIs^27,28^. In agreement with these previous studies, there was a significant association of *CXCL13* and TLS with high inflammation in our cohort. Further, recent studies have shown that TLS promotes recruitment of lymphocytes to the tumor microenvironment by expressing chemokines such as *CXCL10, CCL19* and *CCL21*^31^. Consistent with previous findings, these chemokines were all upregulated in high inflammation MIBCs in our cohort. Taken together, this could explain improved outcome in patients with highly inflamed phenotype.

TLS has been closely associated with the recruitment, activation and proliferation of B cells^32^. Consistent with this finding, our analysis has identified upregulation of numerous B cell markers in MIBC with high inflammation. Depending on the B-cell subtype, mode of activation and the microenvironment, these cells can either contribute to upregulation of T-cell responses or they can exert immunoregulatory functions and participate in the downregulation of T-cell immunity. Recently, CD19 + tumor-infiltrating B-cells were identified as an independent favorable prognostic factor in MIBC and serve as antigen-presenting cells to activate CD4 + T cell in the tumor microenvironment^33^. In agreement with these findings, we found upregulation of *CD19* and *CD4* in high inflammation MIBC. Further, antigen processing and presentation was one of the most enriched biological processes distinguishing high from low invasive front inflammation in our study.

The current findings add to a growing body of literature characterizing the immune genes and signaling pathways in MIBC utilizing the TCGA MIBC cohort. In 2017, Ren R. et al.^34^ performed transcriptomic analysis of immune genes in the TCGA MIBC cohort in association with MIBC molecular subtypes (TCGA subtypes 2014)^34^. They found an increased expression of immune-associated genes in Cluster IV and underactive immune environment in Cluster I, which could be of potential significance for immunotherapies^34^. The study also identified responses to IFN-γ as one of the most common overrepresented pathways in the immune transcriptome of MIBCs and investigated it in the context of TCGA molecular subtypes. Zhou et al. explored 29 immune gene signatures in MIBC TCGA data to identify 3 MIBC immune subtypes characterized by high, medium and low immune gene expression signatures^35^. The group then used a prediction algorithm to estimate the level of immune cell infiltration, stromal content, and tumor purity of each MIBC. They found that immunity high subtype had a high level of immune infiltration, proportion of specific tumor infiltrating lymphocytes, HLA abundance, PD-L1 expression levels, were more likely to respond to ICIs and had relatively better clinical outcomes^35^. Lastly, a pan-cancer study based on TCGA data identified 6 immune subtypes for 33 non-hematologic tumors, including MIBC^36^. They also used prediction algorithms to estimate immune cell content from the TCGA multi-omics data among the 6 immune subtypes to identify immunogenomic features that were predictive of outcome. This study found the immunosuppressed subtype had the worst prognosis while IFN-γ dominant and inflammatory subtypes had the most favorable prognosis. Both studies comprehensively studied the immunogenomics characteristics of MIBC TCGA cohort and suggested a prognostic value for immene signature based molecular subtyping of MIBC. This is distinct from our current study, which characterized the immune transcriptome signatures associated with the degree of inflammation in MIBC as seen on histopathological review.

In this study, we used retrospective FFPE material from a small cohort and the TCGA dataset, which has been generated from frozen bladder tumors. Another possible difference between the cohorts is in the morphological features used to define inflammation. Within the Sunnybrook cohort, five patients received remote intravesical Bacille Calmette Guerin (BCG). It is unclear what (if any) impact this had on the overall immune composition. Despite these limitations, the IRG transcriptional landscape was similar across the two cohorts. The findings reported in the Sunnybrook cohort are based on a limited sample size. Therefore, further research with larger sample sizes are needed to validate the immune signature and more experimental research is needed to fully elucidate the biological mechanisms underlying inflammation, response to immunotherapy, response to chemotherapy and clinical outcome in MIBC.

We discerned two distinct transcriptomic immune profiles for high- and low- tumor inflammation MIBCs and identified a 149 IRG signature associated with high-tumor inflammation, a subset of which also have potential prognostic utility. The immune profile of high invasive front inflammation was characterized by both upregulation of pro-inflammatory chemokines and immunosuppressive checkpoints. Notably, we found upregulation of IFNɣ-mediated chemokine signature in high inflammation MIBC as well as elevated *PD-L1* expression. These findings were validated in the TCGA dataset. The findings further enhances our understanding of the tumor immune milieu and supports its clinical importance for predicting prognosis and immunotherapeutic responsiveness in MIBC, which warrants further investigation.

## Materials and methods

### Sunnybrook cohort clinicopathological variables

Institutional approval was obtained from the Sunnybrook Health Sciences Centre research ethics board for the study (REB 187-2016) and the need for written informed consent for this retrospective study was waived under the approval of the IRB. All procedures were performed in accordance with the Declaration of Helsinki and its later amendments or comparable ethical standards.

Retrospective formalin-fixed paraffin-embedded (FFPE) cystectomy specimens from 40 high-grade muscle-invasive urothelial carcinomas treated at Sunnybrook Health Sciences Centre were included in the current study, 20 with high inflammation and 20 with low inflammation (see definition below). This is a subset of randomly selected cases from a larger cohort of 235 cases characterized in our previous study^6^. Clinicopathological and follow-up data (Table 1) was obtained according to protocols approved by the Research Ethics Board of Sunnybrook Health Sciences Centre. All tumors had predominant urothelial histology. Event-free survival (EFS) was defined as the time from surgery to the following defined events: recurrence, death or palliative care. Subjects who did not experience an event were censored from date of last follow-up. MIBC patients in this cohort did not receive adjuvant or neoadjuvant immune checkpoint inhibitors. Two patients with high and three with low inflammation received neoadjuvant gemcitabine and cisplatin. Five patients received prior intravesical BCG: two in the high invasive front group and three in the low invasive front cohort. All H&E slides were reviewed by a urological pathologist (MRD) for the following: histology, grade (2004 WHO/International Society of Urological Pathology), stage (AJCC/TNM), LVI, margin status (a positive ST margin was defined as tumor extending to ink), carcinoma in situ (CIS) and lymph node metastases.

### Assessment of inflammation in the Sunnybrook cohort

For each case, slides containing urothelial carcinoma were selected and inflammation was semiquantitatively graded by two pathologists (AH/MRD) using the four-point system developed by Klintrup-Makinen^37^ as previously described^6^. Inflammation was assessed at both the invasive front (defined as the deepest interface of carcinoma with stroma) and centrally within the tumor and assigned a score of 0, 1, 2 or 3 at each site. All inflammatory cells were included in the assessment. At the invasive front, inflammation was scored as follows: 0, none; 1, patchy infiltrate; 2, bandlike infiltrate; and 3, prominent with destruction of urothelial carcinoma cells. Only the inflammatory component in contact with and directly adjacent to urothelial carcinoma was scored. Within the tumor, inflammation was scored as follows: 0, absent; 1, scattered inflammatory cells; 2, small aggregates of inflammatory cells; and 3, dense intratumoral inflammation. The scores were averaged across both components and subsequently dichotomised as low (score 0 or 1) and high (score 2 or 3). The presence/absence of tertiary lymphoid structures (TLS) were assessed using published criteria^30^. We defined TLS as well defined clusters of lymphocytes including those with germinal centres. Loose lymphoid collections were recorded as lymphoid aggregates.

### Sunnybrook cohort RNA extraction and NanoString Gene expression profiling

RNA was extracted from macrodissected FFPE tissue Sections (4 to 10 sections per case, each 5 µm thick) using the High-Pure FFPET RNA Isolation Kit (Roche Diagnostics, Indianapolis, IN, USA), according to the manufacturer's protocol. RNA was quantified with the Thermo Scientific Nanodrop 1000 (Thermo Fisher Scientific, Wilmington, DE). RNA profiling was performed with 250 ng (quantified using NanoDrop-1000, Thermo Fisher Scientific, Waltham, Massachusetts, USA) of RNA using the NanoString nCounter Human V.1.1 Pan-Cancer Immune Profiling Panel (NanoString Technologies Inc, Seattle, Washington, USA), according to the manufacturer’s instructions.

Raw gene expression data was analyzed using NanoString's software nSolver V.4.0 with the Advanced Analysis 2.0 plugin. Data normalization was performed using internal negative control probes, synthetic positive controls and 36 selected housekeeping genes that were identified using the nSolver normalization module, which uses the geNorm algorithm (https://genorm.cmgg.be/). Data was normalized using geometric mean, log2-transformed and then used as input for further analysis. KMeans consensus clustering was performed using Pearson distance and average linkage in GenePattern (Broad Institute of MIT and Harvard, http://www.broad.mit.edu/cancer/software/genepattern). For each value of k from 2 to 10, 1000 iterations were performed. The consensus cumulative distribution function (cdf) and Δ area plots were examined and k = 2 was determined to be the ideal model. Unsupervised hierarchical clustering analysis using Euclidean correlation was performed using the R package ComplexHeatmap version 1.12.0. *P* values were adjusted using the Benjamini-Hochberg (BH) method to control the false discovery rate (FDR). Statistically significant, differentially expressed genes (DEGs) were defined as those with expression levels corresponding to a log2 ratio > 1 or <  − 1 and BH q < 0.05. Functional and pathway enrichment analysis was performed using g:Profiler (V.e94_eg41_p11_9f195a1) with a Benjamini-Hochberg (BH-FDR) multiple testing correction method applying significance threshold of 0.05^38^.

### TCGA cohort

Level three RNA sequencing (RNA-seq) data of The Cancer Genome Atlas (TCGA) Urothelial Bladder Carcinoma and corresponding clinical information were downloaded from The Genomic Data Commons Data Portal (https://portal.gdc.cancer.gov/) and log2-transformed prior to analysis. Accession numbers and HGNC IDs were obtained for each ENSEMBL Gene ID in the TCGA database using the BioMart online tool (www.ensembl.org/biomart). The 730 genes represented on the NanoString’s Pan-Cancer Immune Profiling Panel were then selected for downstream analysis (by matching Accession numbers and/or HGNC IDs). Data regarding immune infiltrates was obtained from publication by Robertson et al.^39^ Table S2: Supplemental data was downloaded from Robertson et al.^39^, data in tab S2.4 Immune infiltrate^39^ was dichotomized (present/absent) and matched to RNA-seq data using TCGA_IDs.

### Statistical analysis

All statistical analyses were performed with SPSS 24.0 (IBM Corporation, Armonk, New York, USA). Univariate Cox proportional hazards analysis was used to correlate gene expression with EFS. BH corrected two-sided P-values less than 0.05 were considered statistically significant.

## Supplementary Information

Supplementary Information.

## Data Availability

Data are available from the corresponding author on reasonable request.
