# Supplementary material for: Upregulation of IFNɣ-mediated chemokines dominate the immune transcriptome of muscle-invasive urothelial carcinoma
Source: Sci Rep. 2022 Jan 13;12:716. doi: 10.1038/s41598-021-04678-7 (PMC8758674; doi:10.1038/s41598-021-04678-7)
Supplement: Supplementary file 1 — Supplementary Information. [file 41598_2021_4678_MOESM1_ESM.docx]

**
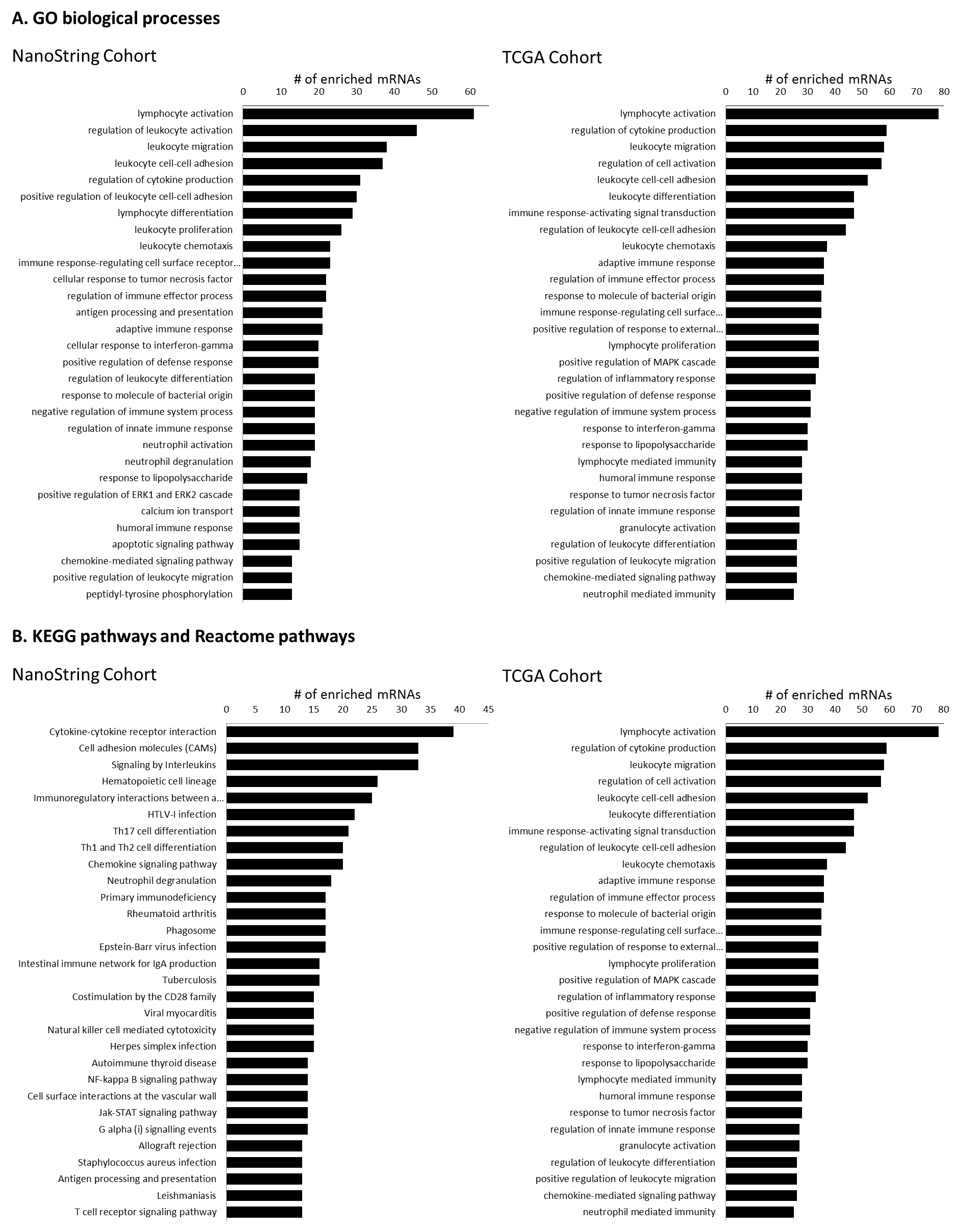
**

**Supplemental Figure 1.** Pathway enrichment annotations from g:profiler for differentially expressed genes between high- versus low- inflammation muscle invasive urothelial carcinomas in NanoString Sunnybrook cohort and carcinomas with- versus without- immune infiltrates in the TCGA cohort.
